# Aurora Kinases and Potential Medical Applications of Aurora Kinase Inhibitors: A Review

**DOI:** 10.14740/jocmr2295w

**Published:** 2015-08-23

**Authors:** Paschalis Gavriilidis, Alexandros Giakoustidis, Dimitrios Giakoustidis

**Affiliations:** a Department of Surgical Oncology, Theageneio Anticancer Hospital, Thessaloniki, Greece; b Department of Transplantation Surgery, Hippokrateion University Hospital, Thessaloniki, Greece

**Keywords:** Serine/threonine kinases, Aurora kinases A, B and C, Aurora kinase inhibitors

## Abstract

Aurora kinases (AKs) represent a novel group of serine/threonine kinases. They were originally described in 1995 by David Glover in the course of studies of mutant alleles characterized with unusual spindle pole configuration in Drosophila melanogaster. Thus far, three AKs A, B, and C have been discovered in human healthy and neoplastic cells. Each one locates in different subcellular locations and they are all nuclear proteins. AKs are playing an essential role in mitotic events such as monitoring of the mitotic checkpoint, creation of bipolar mitotic spindle and alignment of centrosomes on it, also regulating centrosome separation, bio-orientation of chromosomes and cytokinesis. Any inactivation of them can have catastrophic consequences on mitotic events of spindle formation, alignment of centrosomes and cytokinesis, resulting in apoptosis. Overexpression of AKs has been detected in diverse solid and hematological cancers and has been linked with a dismal prognosis. After discovery and identification of the first aurora kinase inhibitor (AKI) ZM447439 as a potential drug for targeted therapy in cancer treatment, approximately 30 AKIs have been introduced in cancer treatment.

## Introduction

Living organisms grow, regenerate and inherit their genetic material with the help of the cell cycle process. As a result of the ordered events of the cell cycle, two daughter cells appear with two exact halves of genetic material. The principal characteristic of cell division is precise division of genetic material. The replication of chromosomal DNA takes place in the S phase of the cell cycle. Segregation of chromosomes and maintenance of diploid chromosome content is completed during mitosis; vast numbers of proteins precisely tune and smoothly regulate this process. Mitotic kinases are essential regulators of mechanisms of protein localization, proteolysis and phosphorylation and contribute for a smooth progression through mitosis [1, 2]. During mitosis, microtubules, the building material of the mitotic spindle and the centrosome, play an important role in regulating tubulin dynamics [3]. Any errors in mitotic signaling pathways can lead to uncontrolled proliferation, one of the principal characteristics of tumors.

Intervention and regulation of mitotic events are the pillars of contemporary chemotherapy. The main flaw of the current antimitotic drugs is that they target simultaneously proliferating cancer cells and non-proliferating healthy cells leading to side effects [3, 4]. Therefore, researchers are trying to identify more specific mitotic targets. Lately, cyclin-dependent kinases, survivin, polo-kinase and aurora kinases (AKs) have been proposed as targets for anticancer therapy [5-7].

**Figure 1:**
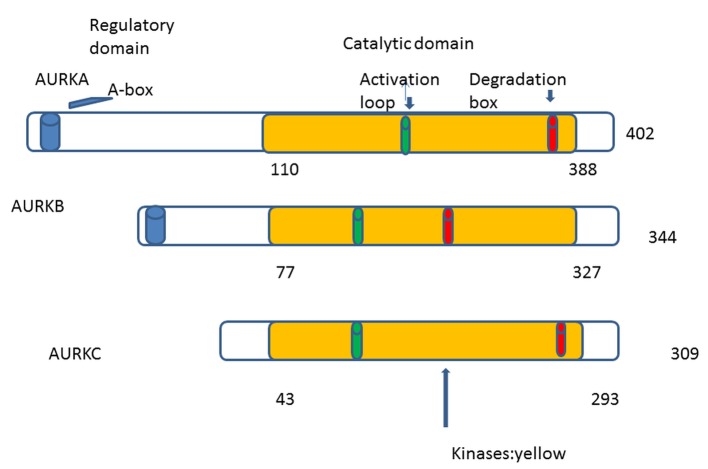
AKs contain mainly two domains: 1) NH2-terminal regulatory domain (white), 2) COOH-terminal catalytic domain (yellow). The three auroras A, B, and C share great homology in the catalytic domain. Phosphorylation at threonine within the activation loop is necessary for kinase activity.

The first human homologue of AKs was described by Bischoff and Zhou in 1998 [4, 8]. So far in human cells, three AKs were identified: A, B and C [9]. AKs structure has been conserved during eukaryotic evolution [1]. They comprise two domains: the NH2-terminal regulatory domain and the COOH-terminal catalytic domain. All three share great homology in the catalytic domain but differ in the regulatory [10, 11] (Fig. 1). In 2004, the first patient was treated with aurora kinase inhibitor (AKI) (PHA-739358) [12]. The translational period was only 8 years. Comparing with the translational period of other targeted therapies, we can remark a real progress.

## Aurora A

Each AK maps in a different chromosome, occupies particular subcellular localizations, turns over and regulates different cell cycle events.

Aurora A maps to chromosome 20q13.2; its activity is synchronized by phosphorylation and dephosphorylation as well as by its expression and degradation. Aurora A is degraded or inactivated in the G_1_ phase of the cell cycle and in contrast to G_2_/M phases, it expresses its maximal activity. It is persistently expressed and strictly regulates mitotic events from mitotic entry through mitotic exit including centrosome maturation, centrosome separation, bipolar spindle assembly, chromosome alignment and cytokinesis [13-15].

Aurora A is considered a bona fide proto-oncogene and the interest of researchers was intensified when first aurora A was found to be overexpressed in human breast cancer cell lines and so named breast tumor activated kinase (BTAK) and in primary colorectal carcinoma biopsies. Consequently, they observed the ability of overexpressed aurora A to transform rodent cells NIH3T3 and rat1 and to provoke tumorigenesis in nude mice [16-19].

Goepfert et al observed in early stages of oncogenesis of the rat breast cancer model an overexpression of aurora A and centrosome amplification [17]. In 2007, Nishida et al showed that overexpression of the aurora A gene is related with chromosomal instability in colorectal cancers [18]. It has also been reported that overexpression and amplification of aurora A in hepatocellular and gastric cancer is a marker of aneuploidy and dismal prognosis [19, 20].

Normal growth rate of cells, cell viability and oncogenesis depend on interactions and balances between aurora A and tumor suppressor gene products such as Chfr, BRCA1, and p53 [21-24]. Chfr is a known tumor suppressor and a mitotic checkpoint protein that confirms chromosomal stability by interacting and influencing expression of aurora A [21]. Ouchi et al showed that aurora A connects to BRCA1 and phosphorylates Ser 308. They also suggested that disrupted regulation of G_2_/M transition by BRCA1 and aurora A predisposes to carcinogenesis [22]. Aurora A is an important regulatory constituent of the p53 pathway and interacts with it at multiple levels. It is overexpression inhibits the p53 suppression function, which facilitates oncogenesis [23]. Moreover, aurora A interacts with p53 and influences its suppression function by at least two ways: firstly it directly phosphorylates p53 at Ser 315 enabling mdm2 mediated degradation of p53 in tumor cells and secondly it inactivates its transcriptional activity by phosphorylating p53 at Ser 215 [23, 24]. It is suggested that aurora A, as a constituent to the Ras/Raf/MEK/ERK/Map kinase pathway of a human carcinogenesis, might influence the growth rate of cancer cells, and also stimulates telomerase activity and human telomerase reverse transcriptase by up-regulation of c-myc23 and promotes collagen I-induced cell migration [25]. Therefore aurora A might promise prolonged survival in cancer cells.

Overexpression of aurora A plays a crucial role in the inactivation of apoptosis in cancer cells by the up-regulation of an anti-apoptotic effector NF-kB [26].

## Aurora B

Aurora B maps to chromosome 17q13. Independently and of great similarity in structure and sequence, aurora A and B are totally different in subcellular localizations; their subcellular geography determines independent functions in mitotic events. During mitosis and when prometaphase starts, aurora B leaves the nucleus and relocates to centromeres until metaphase; once anaphase begins, it moves steadily to the midzone and remains at the midbody until the end of cytokinesis [27].

During mitosis, aurora B forms a tight complex with its binding partners and substrates such as: inner centrosome protein (INCEP), borealin and survivin [26]. This complex called chromosomal passenger complex plays an essential role in the establishment of proper kinetochore-microtubule attachments [28, 29]. Aurora B also assists in proper chromosome bio-orientation, accurate chromosomal segregation, regulation of the mitotic checkpoint and cytokinesis [30].

While inhibition of aurora A leads to arrest in mitosis, in contrast inhibition of aurora B inactivates the checkpoints and leads to chromosome misalignment and failed cytokinesis [31]. Owing to this characteristic, aurora B inhibitors have been named as mitotic drivers in a review [32].

Multinucleation and polyploidy are consequences of aurora B overexpression; in addition, these phenotypes are aggravated in absence of p53 [33]. Moreover, overexpressed aurora B causes chromosome insulation in metaphase, defects in chromosome segregation and defects in cytokinesis, so inducing the process of carcinogenesis [34].

Aurora B is overexpressed in hepatocellular, colorectal, breast, renal, lung and thyroid cancer [35-37]. It is reported that overexpressed aurora B is an essential marker related with tumor invasiveness, early recurrence and dismal prognosis [38].

Inappropriate cell movement that underlies tumor invasive and metastatic possibility are promoted by both AKs A and B [39].

Yasen et al [40] studying the role and expression of aurora B and its variants, wild type, splicing variant 1 and 2 in hepatocarcinogenesis and in liver tumors concluded that aurora B splicing variant 2 (SV2) was associated with hepatocellular cancers (HCCs) diagnosed with vascular invasion of hepatic and portal veins. Therefore, there is justified evidence to correlate this variant with dismal prognosis and high rate of recurrence in HCC. They also detected SV2 to be overexpressed in cases of multiple tumor formation, capsular invasion and increased AFP levels. Characteristically, SV2 was not detected in normal hepatic tissues but only in neoplastic tissues. Based on these features, inhibition of aurora B and its variants might provide useful tools for neoadjuvant and adjuvant treatment of the HCC in the near future.

## Aurora C

Aurora C is the less investigated aurora compared to AKs A and B. It maps to chromosome 19q13. It was first discovered in testis where it functions in spermatogenesis [41].

Structurally, it is a chromosomal passenger protein like aurora B and is more similar to it (83%) than to aurora A (71%). It plays an essential role in mitotic events and in particular in centrosome function and is identified to it from anaphase to cytokinesis [41]. Similar to aurora B, its mRNA and protein expression levels peak at G_2_/M phases [41].

Studying the role of aurora C on female mouse meiosis, researchers detected that aurora C inactivation brings about defects such as premature chromosome segregation, chromosome misalignment, abnormal kinetochore-microtubule attachment and cytokinesis in meiosis [42].

It is reported to be overexpressed in HeLa, HepG_2_, MDA-MD-453 and Hu H7 cancer cells [43]. However, there is no enough evidence for its role in carcinogenesis and further research is required to clarify its role in molecular pathways in cancer.

Aurora B and C are closely related and most likely when developed, form a common ancestral aurora B/C of cold-blooded vertebrate [44, 45].

## Potential Oncological Applications

Thus far, many studies found that overexpressed and amplified AKs are tightly associated with a large number of cancers. Due to their participation in mitotic events and influence on important processes of human carcinogenesis such as Ras/Raf/MEK/ERK/MAP kinase pathway, telomerase activity, collagen I-induced cell migration and anchorage-independent growth, they trigger interest for researchers to develop potential inhibitors against them (Table 1).

**Table 1:** Potential Oncological Applications

Aurora kinase inhibitors	Targets	Oncological applications
Tozasertib	Aurora A, B and C	First AKI in clinical use, simultaneous and successive treatment in CML and ALL
PHA-739358 (danusertib)	Aurora A, B, C TrKA-a, Ret, FGR	Phase II in prostate cancer, in combination use with sorafenib in HCC, phase II in multiple myeloma
CYC116	Selective effect on aurora A, B, inhibition of angiogenesis	In combined use with ionizing radiation on ras-mutated colorectal adenocarcinoma, phase II in solid tumors
SNS-314	Pan-aurora kinase inhibitor	Preclinical studies of cell lines and murine models of prostate, colon and breast cancers, phase I in solid tumors
AMG-900	Pan-aurora kinase inhibitor	Preclinical studies of hematological and solid tumors
VE-465	Pan-aurora kinase inhibitor	Antineoplastic activity in preclinical studies of HCC, ovarian cancer and hematologic malignancies
AS703569/R-763	Aurora A, B, C FLT1, FLT3, Abl, Akt, VEGFR3	Anticancer activity in cell culture in cell culture and murine xenograft models of hemopoietic and solid tumors
PF-03814735	Aurora A, B, C, FGFR1, FAK, TrKA, FLT1, MET	Phase I for solid tumors
AT9283	Aurora A, B, Abl, JAK3, T3151 BCR-ABL, wild-type BCR-ABL	Preclinical efficacy in hematologic malignancies
GSK1070916	Aurora B-INCEP, C-INCEP, SIK, FLT1, FGFR1, FLT4	*In vivo* efficacy in xenograft models, phase I in solid tumors
MLN8054	Selective inhibitor of AURKA	Withdrawn from clinical use due to sever somnolence and central nervous system toxicity
MLN8237	Aurora A, B, T3151 BCR-ABL	Seven ongoing phase II studies, one phase III recruiting patients

## Pan-Aurora Inhibitors

### Tozasertib

Tozasertib is a pyrimidine derivative with high affinity to AKs, imatinib-resistant and dasatinib-resistant forms of ABL. Its inhibitory constant values are lower for aurora A 0.6 nM and C 4.6 nM than for aurora B 18 nM [46]. *In-vitro* showed a 100-fold more selectivity for AKs than for a pool of 55 other kinases. *In-vivo* demonstrated inhibitive possibilities of a large number of cancers. In particular, tumor xenograft models regressed and reduced the tumor size by 22-98% [47].

Tozasertib was the first AKI to be used in patients. Its main side effects were reversible neutropenia and anorexia. It was stopped in 2007 because QTc prolongation occurred in one patient. However, ASCO2009 reported its use in patients with chronic myelogenous leukemia (CML) and ALL in simultaneous and successive treatments with dasatinib [46].

Patients with a solid advanced tumor tolerated well tozasertib in doses up to 8 mg/m^2^/h in phase I study [48].

### PHA-739358 (danusertib)

Danusertib is a potent pan-aurora kinase inhibitor; its half maximal inhibitory concentration (IC50) is obviously the smallest for aurora A only 13 nM, the highest for B 79 nM and for C 61 nM. Chemically it is a 3-aminopyrazole derivative [49]. It also inhibits several cancer related tyrosine kinases as well as Abl, Trk-a, fibroblast growth receptor-1 and Ret [49]. This feature of cross-reactivity enhances its activity and broadens its indications in hematological and solid malignancies such as CML, ALL, thyroid, prostate and breast cancer [49]. Danusertib has significant anti-tumor activity in transgenic models with a favorable preclinical safety profile; target organs of danusertib are hemolymphopoietic system, gastrointestinal tract, kidneys and male reproductive organs [49].

Benten et al observed that combination of multikinase inhibitor sorafenib and PHA-739358 caused a virtual growth arrest of hepatocellular cancers. In addition, standard monotherapy dose of PHA-739358 alone was capable of shrinking tumors even after treatment failure under sorafenib [50].

Fraedrich et al showed that danusertib inhibits advanced gastroenteropancreatic neuroendocrine tumor cell growth in association with cell-cycle disruption and induction of apoptosis [51].

Cohen et al [52] in a phase I study treated 56 patients with advanced solid tumors by dose escalating danusertib. Group I consisted of 40 patients and was treated by danusertib alone; the maximum tolerated dose (MTD) was 500 mg/m^2^. Group II consisted of 16 patients and was treated concomitantly with danusertib and granulocyte colony stimulating factor (G-CSF) filgrastim; the MDT was 750 mg/m^2^. In one patient of group II with diagnosis of refractory small cell lung cancer, objective tumor response was observed when he was treated with doses at 1,000 mg/m^2^. Subsequently the dose was reduced to 750 mg/m^2^ when azotemia occurred. They also observed disease stabilization in 43% of patients of group I and 50% of group II. The time period of 23.9 to 52.3 weeks was defined as being prolonged disease stabilization time and was only observed in four patients of group I. Tumor regression by 27% and declination CA-125 by 30% was achieved in a woman with ovarian cancer. The most frequently encountered serious side effects were leukopenia, neutropenia, febrile neutropenia, neutropenic infections, azotemia and pyrexia. They concluded that their results provide ample justification for further clinical trials [52].

Danusertib related renal side effects have been investigated in rats and dogs. In the rat model, tubular nephropathy was noticed at the 600 and 750 mg/m^2^ intravenous (IV) bolus equivalent doses. In repeated dose studies with 6-h IV infusions, an increase in urea and creatinine was observed in rats (but not in dogs) at the MTD 720 mg/m^2^ and was rescindable [52]. In man, an increase in grade 1 and 2 creatinine was seen when the administered dose was > 1,000 mg/m^2^ [52].

Steeghs et al enrolled 50 patients with advanced or metastatic solid tumors in a phase I study; in group I was given danusertib at starting dose 45 mg/m^2^ in 6-h IV infusion scheme, in group II 250 mg/m^2^ (IV) in 3-h scheme. On both groups danusertib was given on days 1, 8, and 15 every 4 weeks. They reported neutropenia as a main adverse effect on both groups. They did not observe partial or complete responses. Overall disease control was achieved only in six of 30 patients (20%) of group of 6-h infusion. Disease stabilized for more than 6 months in four patients in the 6-h scheme and in one in the 3-h scheme. Interestingly, disease stabilization was achieved in one patient with uncontrolled non-small cell lung cancer treated on the 6-h scheme before the study. Finally they concluded that the recommended dose of danusertib for phase II studies is 330 mg/m^2^ in 6-h IV infusion on days 1, 8, and 15 every 4 weeks [53].

Meulenbeld et al [54] studied the single agent efficacy of danusertib in patients with metastatic castration resistant prostate cancer after docetaxel failure. They concluded that danusertib monotherapy is ineffective.

Recently, danusertib proved to be effective on treating the neglected disease African sleeping sickness or human African trypanosomiasis. Ochiana et al demonstrated that danusertib inhibits trypanosomal aurora kinase 1 (TbAUK1) and kills the parasite *in vitro* [55].

### CYC116

CYC116 is a pan-AKI which in preclinical studies demonstrated selective effects on aurora A and B. Its route of administration is oral and overall antitumor effect is based on inhibition of angiogenesis [56, 57].

It demonstrated characteristic activity against cancer models of both cell lines and murine xenografts of thyroid, breast, pancreatic, colorectal melanoma and non-small cell lung cancers [58, 59].

It is also demonstrated synergy when used in combination with chemotherapeutic agents and ionizing radiation [59]. The results of the preclinical study showed that the combined use of the CYC116 with ionizing radiation has stronger anti-tumor results on Ras-mutated colorectal adenocarcinoma than on Ras-wild type cell lines [59].

### SNS-314

SNS is a pan-AKI expressing highest affinity for aurora B (IC50 = 31 nM) followed by aurora A (9 nM) and aurora C (3 nM). It is aminothiazole-derived urea; its anti-tumor effects for cancers of prostate, colon, breast, melanoma and ovary were demonstrated in preclinical studies of cell lines and murine models [60].

One of the principal characteristics of SNS314 is the exhibition of synergy when it is used in combined treatment schemes with other chemotherapeutic agents in colorectal cell lines; moreover with antimicrotubule agents, its synergistic effect is further enhanced [61].

### AMG-900

It is a small molecule pan-aurora inhibitor orally administered and demonstrates characteristically little off-target inhibition [62]. Its anti-proliferative capacities are proved against cell lines of both hematologic and solid cancers. Furthermore, it proved to be effective in cell lines resistant to paclitaxel, AZD-1152, tozasertib and danusertib [62].

### VE-465

VE-465 is an aurora inhibitor related to MK0457. Its antineoplastic activity was confirmed in preclinical studies of culture cells and murine xenograft models of hepatocellular carcinoma, ovarian cancer, multiple myeloma, myeloid leukemia and CML [63, 64].

### AS703569/R-763

This pan-AKI inactivates aurora A, B and C at IC50 values of 4, 4.8 and 6.9 nM respectively. Furthermore, it inhibits oncogenic kinases including fms-related tyrosine kinase (FLT1), FLT3 and Abl [65].

It is an orally administered aurora inhibitor exhibiting effective wide inhibition of Akt, VEGFR3, FLT3, Bcr-Abl, and IGFR [65]. Its anti-proliferative activity was demonstrated in cell culture and murine xenograft model of hemopoietic and solid cancers including biphenotypic leukemia, acute promyelocytic leukemia, acute lymphocytic leukemia, CML, multiple myeloma, colorectal, ovarian, breast and pancreatic cancers [65, 66].

### PF-03814735

PF-03814735 is another pan-aurora inhibitor expressing highest affinity for aurora A and B at IC50 of 6 and 0.8 nM, respectively. Moreover, at higher IC50 values, it inhibits fibroblast growth factor receptor 1 (FGFR1), focal adhesion kinase (FAK), TrKA, FLT1, and MET kinases [67]. A recent phase I study was conducted where 57 patients with solid tumors were enrolled. In arm A, 32 individuals were treated for 5 days with 5 - 100 mg/day; in arm B, 25 individuals were treated for 10 days with 40 - 60 mg/day of 21-day cycles. For the patients of arm A, 80 mg/day was the MTD. In this cohort of patients, two suffered grade 3 and grade 4 febrile neutropenia and one suffered from grade 3 proctalgia. In arm B, 50 mg was the MTD where two patients suffered from ventricular dysfunction and one grade 3 increase of aspartate aminotransferase. The substance was easily absorbed, appeared in 6 h and showed favorable linear pharmacokinetics.

Results wise, stabilization of the disease was observed in 19 patients. Furthermore, tumor reduction was observed in five patients of arm A with stable disease occurring [68].

### AT9283

AT9283 has a wide range inhibitory effect at IC50 of 3 nM. It inhibits both aurora A and B and at IC50 of 110 and 3 nM, it inhibits T3151 BCR-ABL and wild-type BCR-ABL, respectively [69, 70]. Moreover it targets ABL, Janus kinase (JAK), JAK3 and restrains proliferation of BaF3 cells in both T3151 BCR-ABL and wild-type BCR-ABL [69, 70].

In K562 xenograft mouse model of BCR-ABL positive CML has been observed to reduce significantly tumor volume at dose 12.5 mg/kg [70].

In phase I trial where 30 patients with refractory leukemia were enrolled with a dose range of 3 - 162 mg/m^2^/day, dose limiting toxicity (DLT) was not observed up to dose 72 mg/m^2^/day. Only in one case at a dose of 12 mg/m^2^/day tumor lysis syndrome was observed. The most common developed DLTs were mucositis, septicemia and pneumonia [71].

### GSK1070916

GSK1070916 selectively inhibits aurora B-INCEP and aurora C-INCEP. It interacts with tyrosine kinase with immunoglobulin-like and EGF-like domains (TIE2), salt inducible kinase (SIK), FLT1, FGFR1 and FLT4 [72]. After testing its *in-vitro* activity on 161 tumor cell lines, inhibition of cancer lines at IC50 of 8 nM without showing any remarkable anticancer activity on non-proliferating HUVEC cells was observed [73].

*In-vivo* efficacy of the GSK1070916 has been examined in several xenograft models: in MCF-7, colo205 and H460. Stabilization of the process and partial or complete regression in K562, HCT116, HL60 and A549 models was observed [73].

## Selective AKIs

### MLN8054 (millennium)

This substance is a highly selective inhibitor of aurora A. It acts not by degradation or down-regulation of aurora A but inactivates its phosphorylation. It is > 40 folds more effective inhibitor of aurora A than of aurora B. However at higher concentrations it inhibits also aurora B. Abnormal DNA content in cells treated with millennium is observed [74].

Remarkable tumor growth inhibition occurred in tumor xenograft models treated with MLN8054. However the substance was withdrawn from clinical trials due to severe side effects including severe somnolence, central neurocognitive changes and central nervous system toxicity. This phenomenon was explained by the structural similarity of the MLN8054 to the benzodiazepines and associated affinity to gamma-aminobutyric acid a1 receptor (GABA a1) [75, 76].

### MLN8237 (alisertib)

Millennium invented a second generation selective inhibitor of aurora A, the 5-H-pyrimidol[5,4-d] [2] benzazepine MLN8237 due to the demonstrated side effects of the MLN8054. In preclinical studies, inhibition of aurora A was achieved with an IC50 value of 1.2 nmol/L, while aurora B inhibition was achieved with a value of 396.5 nmol/L [9]. The demonstrated most common DLTs in phase I studies were thrombocytopenia, neutropenia, stomatitis and diarrhea [77, 78]. *In-vivo* antitumor activity of the drug in various xenograft tumor models was observed [79]. The substance has been found to be active against T3151 BCR-ABL and BCR-ABL mutated cells in culture [80]. MLN8237 has been examined on pediatric cancer cell lines such as glioblastoma, Ewing sarcoma, neuroblastoma, rhabdomyosarcoma, AML and ALL. The highest efficacy was demonstrated against the ALL cell lines and the least on rhabdomyosarcoma [81].

## Combined Use of AKIs With Histone Deacetylase (HDAC) Inhibitors

Recently patients with advanced phase CML treated with BCR-ABL tyrosine kinase inhibitor imatinib, have developed resistance to treatment [82]. The cause of this phenomenon is based on point mutations in the kinase domain of BCR-ABL [83]. Researchers tried to find a solution to the problem by combining AKI tozasertib with HDAC inhibitors pracinostat and/or vorinostat. They concluded that the combination of AKI tozasertib and HDAC pracinostat and/or vorinostat is highly effective against BCR-ABL cells [84].

## The Role of AKs in GIST and Head and Neck Cancers

AK overexpression was identified as a poor prognostic factor for GIST recurrence [85]. Moreover aurora A is significantly overexpressed in non-gastric GISTs than in gastric.

Lagarde et al demonstrated that AK A is strongly associated with GIST tumors with high metastatic potential [86]. Either aurora A measured by immunohistochemistry or by qRT-PCR both confirmed it as an independent prognostic factor for GIST recurrence [85, 86].

An essential prerequisite for oncogenesis in squamous-cell cancer head and neck cells (SCCHN) is the build-up of aurora A in the nucleus [87]. Using immunohistochemistry (IHC) and qRT-PCR, researchers measured aurora A mRNA and aurora A protein expression in 66 head and neck cancers and healthy squamous epithelium. They concluded that decreased disease free survival and overall survival was related to increased levels of aurora A mRNA [88]. Another retrospective study reported overexpression of aurora A mRNA in laryngeal cancer [89].

Qi et al demonstrated that aurora B overexpression in oral cancer is related with increased multinuclear cells, cellular proliferation and lymph node metastases [90].

Phase I trial patients with advanced solid tumors were treated with MLN8054. From the cohort of head and neck tumors, only a few of them demonstrated stabilization of the disease [75, 78].

New therapeutic strategies are investigating the combined use of the EGFR inhibitor cetuximab, AKIs and radiotherapy [91]. Simultaneous use of cetuximab and the AKI R763 has shown inhibition of cell growth in SCCHN cell lines [92]. It is also reported that overexpression of aurora A and EGFR are dismal prognostic factors [92].

## Conclusions

Discovery of AKs provides a new concept to comprehend cell cycle events and especially mitosis which are affected on the beginning of carcinogenesis. Overexpression and/or amplification of AKs is found in a wide range of hematological and solid cancers. Aberrant auroras can lead to genomic instability and subsequently to uncontrolled cell proliferation which is the hallmark of cancer and the prerequisite for emerging accrual mutations which enable cancers to develop resistance to chemotherapeutic agents. Tumor cell sensitivity is highly dependent on intact cell cycle checkpoints. AK inhibition expresses a natural degree of selectivity to cancer cells depleted of a certain mitotic checkpoint proteins and non-functional p53 rather than to healthy tissues. The above feature promises more effective AKIs and fewer serious adverse effects.

## Abbreviations

AK: aurora kinase
AKI: aurora kinase inhibitor
HDAC: histone deacetylase
